# Development and Validation of Psychometric Properties of the 10 IB Learner Profile Instrument (10IBLP-I): A Combination of the Rasch and Classical Measurement Model

**DOI:** 10.3390/ijerph18126455

**Published:** 2021-06-15

**Authors:** Miftahuljanah Kamaruddin, Mohd Effendi Ewan Mohd Matore

**Affiliations:** Research Centre of Educational Planning and Policy, Faculty of Education, Universiti Kebangsaan Malaysia, Bangi 43600, Selangor, Malaysia; jannah.kamaruddin96@gmail.com

**Keywords:** instrument development, 10 IB learner profile instrument (10IBLP-I), psychometric, Rasch measurement model, confirmatory factor analysis, validity and reliability

## Abstract

Background: The International Baccalaureate Middle Years Programme (IBMYP) aims to produce a holistic transformation with creative and critically minded students. However, very little attention has been paid to the development of an instrument to measure the IB learner profile with good psychometric properties. Purpose: This study aims to develop an instrument with good psychometric properties, based on the Rasch measurement model and confirmatory factor analysis. Methods: The study consists of two phases of pilot and field studies involving 597 year four students from IBWS MOE. Results: The findings from the Rasch measurement model analysis have shown that 54 items meet the criteria of the item fit, unidimensionality, and reliability index. Meanwhile, confirmatory factor analysis found that 44 items have shown a valid item fit index. Conclusions: The combination of both analyses has shown the strength of 10IBLP-I psychometric properties that cover the aspects of validity and reliability. The findings also provide an implication to the theory, with empirical evidence that the IB learner profile consists of 10 constructs. Besides, the evidenced 10IBLP-I comprises good psychometric properties, which can be used to measure the level of IB learner profile among IBWS MOE students to assess the effectiveness of the implementation of IBMYP in Malaysia.

## 1. Introduction

In facing fast-growing and uncertain global challenges, the fourth target in the sustainable development goals (SDG) focuses on developing superior human capital that can be a driver for the nation’s progress and development. This can be achieved through global citizenship education that trains students to understand and respect each other’s differences, which can contribute to world peace. In the face of the challenging global competition, the younger generation should be equipped with knowledge, skills, values, and attitudes, so that they are always proactive, able to adapt quickly, and subsequently able to decide and resolve problems [1]. In line with this aspiration, the MOE also focuses on the culturalization of higher-order thinking skills (HOTS) or Kemahiran Berfikir Aras Tinggi (henceforth KBAT) among students, to produce intelligent, creative, and innovative human capital to meet the 21st century challenges, so that the nation can compete in the world.

Towards realizing this global citizenship education, the International Baccalaureate (IB), established in 1968, has been shown to provide holistic education through multicultural understanding and international mindedness, so that they can think critically and independently [1,2,3]. The International Baccalaureate Middle Years Programme (IBMYP) is one of the world’s recognized IB learning programs and has been implemented in 5556 schools worldwide [4]. In Malaysia, IBMYP was introduced by the MOE in the Malaysian Education Development Plan 2013–2025 towards a holistic and effective educational transformation, and resulted in creative and critically minded students. IBMYP has been implemented in 10 Malaysian government secondary schools from 2013, comprising various backgrounds, towards ensuring program feasibility in all types of schools. These following 10 schools are known as International Baccalaureate World School (IBWS) MOE: (1) the Malay College Kuala Kangsar, Perak; (2) Tunku Kurshiah College, Negeri Sembilan; (3) Sekolah Menengah Sains Tengku Muhammad Faris Petra, Kelantan; (4) Sekolah Menengah Kebangsaan Agama Sheikh Abdul Malek, Terengganu; (5) Sekolah Menengah Kebangsaan Putrajaya Presint 9(2), Federal Territory of Putrajaya; (6) Sekolah Menengah Kebangsaan Sultanah Bahiyah, Kedah; (7) Sekolah Menengah Kebangsaan Dato’ Sheikh Ahmad, Perlis; (8) Sekolah Menengah Kebangsaan Seri Tualang, Pahang; (9) Sekolah Menengah Kebangsaan Sungai Tapang, Sarawak; and (10) Sekolah Menengah Kebangsaan Pantai, Federal Territory of Labuan [5].

The effectiveness of the implementation of the IBMYP approach can be assessed based on the level of IB learner profile, namely, inquirers, knowledgeable, thinkers, communicators, principled, open-minded, caring, risk-takers, balanced, and reflective [6]. These 10 IB learner profile attributes can be applied through the implementation of teaching and learning practices, using the IBMYP approach over five years of schooling [7]. The application of 10 IB learner profile attributes is in line with the 21st century learning outcome, which is to enhance and cultivate thinking skills towards creative, innovative, and critical thinking. Students who have the IB learner profile are also independent learners, knowledgeable, inquirers, caring, international-minded, understanding, and respectful of each other’s differences, which can contribute to world peace. Thus, this study aims to develop a good psychometric 10 IB learner profile instrument (10IBLP-I), which is deemed useful not only for IBWS MOE students to self-assess their IB learner profile, but also as an observational measurement tool for teachers to evaluate and monitor the inculcation of the students’ IB learner profiles. The data collected can be used by schools and teachers to support students and instill the IB learner profile among them.

## 2. Research Background

Literature review at the international level has shown numerous studies related to the IB learner profile with other variables. However, these studies are more inclined towards the Diploma Program (DP), which is a pre-preparatory program to further studies at the university level. For example, Poole [8], conducted a case study to explore the methods and approaches of art teacher, Sophie who is an expatriate in interpreting and applying the IB learner profile for IBDP students. Meanwhile, Wells [9] carried out a study by researching the reflection of the IBMYP and DP students to describe their IB learner profile characters and the extent to which these characters can contribute to international mindedness among students. Besides, Gardner-McTaggart [10] conducted an exploratory study on leadership practices among school leaders, through critical phenomenological aspects, by incorporating the IB learner profile application and global citizenship education. In addition, Weiss [11] performed a descriptive study involving 24 research participants to determine teacher perspective towards the IB learner profile attributes. The results showed that teachers considered caring as the most obvious attribute to assess, while balanced was the most difficult attribute to be assessed.

Accordingly, the measurement of 10 IB learner profile attributes among IBWS MOE students requires the development of an instrument with good psychometric properties, such as construct validity and high reliability. However, the existing IB learner profile instruments, developed by Walker et al. [12] and Bryant et al. [13], only involve four attributes out of the overall 10 attributes of the IB learner profile, namely, knowledgeable, inquirers, caring, and open-minded, and this has become a limitation in extant research. In Malaysia, research conducted by the MOE to measure the level of IB learner profile in assessing the effectiveness of IBMYP implementation in 10 IBWS MOE schools in 2015 and 2018 did not use instruments with empirical evidence regarding the reliability and validity of the IB learner profile instrument [14,15]. Besides, to date, no empirical studies in Malaysia have built an IB learner profile instrument, although the implementation of IBMYP in Malaysia is not only limited to schools under the MOE, but also schools under other agencies, such as MARA Junior Science College and private schools. MARA is the Malay acronym for the People’s Trust Council, which was originally set up to drive development and provide financial assistance to Malays. It also offers overseas scholarships and operates junior colleges across Malaysia. Thus, an instrument with the overall 10 attributes of the IB learner profile must be developed to ensure that the IB learner profile level among IBWS MOE students can be measured and the effectiveness of the IBMYP implementation in 10 IBWS MOE schools can be assessed.

Therefore, the study aims to develop an instrument with good psychometric properties, based on the Rasch measurement model and confirmatory factor analysis, to measure the level of IB learner profile among IBWS MOE students. Hence, the research objectives are as follows:

(1) To develop the 10 IB learner profile instruments (10IBLP-I) among IBWS MOE students;

(2) To examine the psychometric properties of the 10IBLP-I developed, based on the Rasch measurement model and confirmatory factor analysis.

### 2.1. International Baccalaureate Program

International Baccalaureate (IB) is a recognized and prestigious program at the international level, conducted by the International Baccalaureate Organization (IBO). In the beginning, the implementation of IB was aimed at fulfilling the need of students who had to follow their parents to migrate to another country, as well as introducing international understanding [16]. This program entails a special pre-preparatory program for entering internationally recognized universities, whilst providing an appropriate and inclusive academic curriculum to students. The main goal of the program is to develop the potential of globally minded students in creating a better and harmonious world and environment. The program also trains students to be more active, humane and inculcates their interest in lifelong learning. Besides that, understanding and tolerance towards various cultures is also the main focus of this program to shape international mindedness among students [4].

IB offers a comprehensive international curriculum through the concept of the education continuum, which includes four programs that differ from as early as three years old to pre-university age, as follows [4]:

i. Primary Years Programme (PYP) offers learning for children aged three to 12 years old. The implementation of the PYP focuses on developing and cultivating the attribute of inquirers among students, which is not only limited in the scope of learning in the classroom, but also through the development of the outside world;
ii. Middle Years Programme (MYP) or IBMYP provides a comprehensive and challenging learning framework for students aged 11 to 16 years old. IBMYP trains and encourages students to be reflective and critical-minded through an understanding of the development of the outside world beyond the scope of the traditional and conventional learning curriculum;
iii. Diploma Program (DP) is a preparatory program for students to pursue their studies at local and foreign universities. DP provides a balanced and challenging learning platform in the aspect of academics, focusing on the international element towards producing outstanding individuals in the academic field, and those who are able to face challenges in the working environment;
iv. Career-related Programme (CP) is a program that targets school leavers as an alternative option for those who intend to continue to venture into career fields. CP can help students prepare before venturing into career fields, through learning experiences that include the vision and principles of IB.

### 2.2. IB Learner Profile

The uniqueness of IB is that this program trains younger generations who are knowledgeable, inquirers, caring, and international-minded. Apart from cognitive development, through the application of HOTS, IB also opens up opportunities for students to build well-being, encompassing social, emotional, and physical aspects [17]. IB introduced the IB learner profile as an action plan for cultivating international-mindedness through 10 attributes, namely, (1) inquirers, (2) knowledgeable, (3) thinkers, (4) communicators, (5) principled, (6) open-minded, (7) caring, (8) risk-takers, (9) balanced, and (10) reflective [6]. Next, these 10 attributes can build the character of each student who attends the IB program to be responsible, tolerant, and to contribute to the harmony of their surroundings [17].

The effectiveness of the IBMYP approach can be assessed based on the application of 10 IB learner profile attributes among students in 10 IBWS MOE. These 10 attributes are a must-have value in students, to shape individual responsibility for the local, global, and national communities and contribute to world peace. The operational definitions for the 10 IB learner profile attributes based on the [6] are as follows:

The first attribute of the IB learner profile is inquirers, which means that students formulate and utilize global understanding and knowledge in ventured areas on new issues and ideas with local or global interests. Next, the second IB learner profile attribute of thinkers means that students use creative and innovative skills in analyzing and taking action against complex problems. Students also take the initiative in making any ethical recommendations and decisions.

The third attribute of the IB learner profile is communicators, which means that students demonstrate confidence and creativity in many ways, by mastering more than one language. Students are also able to collaborate more effectively, and listen to individual and group views carefully. Subsequently, the fourth IB learner profile attribute of principled means that students always act with honesty and trust, with a strong sense of equality and justice, as well as respect for the rights of others. Students are also responsible for the actions they perform and the consequences of their actions.

The fifth attribute of the IB learner profile is open-minded. Through the application of this attribute, students will respect their own culture and history, as well as the values and traditions of other communities. Students can also accept the views of others and advance themselves based on their learning experiences. Meanwhile, the sixth IB learner profile attribute of caring refers to the sense of sympathy, compassion, and mutual respect shown by the students. They are also ready and committed to providing services and making positive changes in daily life as well as in the surrounding world.

The seventh attribute of the IB learner profile is knowledgeable, which refers to the development of students in applying global understanding and knowledge in ventured areas on new issues and ideas with local or global interests. The eighth IB learner profile attribute of risk-takers means that students take an approach about something uncertain by a way of thinking forward. Students also perform their job independently, and work together to explore new ideas and innovative strategies. Next, students are likewise intelligent and resilient in facing the available opportunities and challenges.

The ninth attribute of the IB learner profile is balanced. Through the application of this attribute, students can understand the importance of balance in various aspects of life, either intellectually, physically, or emotionally, to achieve well-being goals for themselves and others. Students can also identify their dependence on others and the world. The final IB learner profile attribute is reflective. The application of this attribute can help students examine the world, ideas, and their own experiences. They can also strive to appreciate their potential and strength, as well as improving weaknesses to support learning and personality development.

### 2.3. Underlying Theories of IB Learner Profile

Based on a systematic review by Bullock [18], the 10 IB learner profile attributes were categorized into four constructs, namely, cognitive, conative, affective, and social. Firstly, the cognitive construct comprises the attributes of knowledgeable, thinkers, and reflective. This construct represents a cognitive process that requires a deep understanding of learning and knowledge through the development of the HOTS concept and application. The underlying theories of the cognitive construct are the cognitive development theory [19], cognitive learning theory [20], Kolb’s cycle learning theory [21], and Bloom’s taxonomy [22]. Next, the conative construct comprises the attributes of inquirers and principles. Conative or personal refers to self-efficacy towards metacogynist exploration that builds student awareness of learning. The goal orientation theory [23], Maslow’s hierarchy needs theory [24], and the self-determination theory [25] are the underlying theories of this construct.

Meanwhile, caring, risk-takers, and balanced are the attributes for the affective construct with the underlying theories of the psychosocial theory [26], ecological theory [27], and emotional intelligence theory [28]. The affective construct or emotional skill refers to the quality of individuals who build confidence and well-being. Finally, the attributes of communicators and open-minded are categorized into the social construct. This construct is also known as a cultural construct that emphasizes collaborative networks in contributing to local communities. Appreciation to cultural diversity and differences in opinion can be valued by each individual. Among the underlying theories of this construct are the social learning theory [29], ecological theory [27], and cultural dimension theory [30].

### 2.4. Past Studies on the Development of Instruments for IB Learner Profile

Walker et al. [12] had developed and validated the International Baccalaureate learner profile questionnaire (IBLPQ) in the year 2015. The IBLPQ was the first instrument developed to measure the IB learner profile by Walker et al. [12]. However, this instrument was only developed to measure 4 out of 10 attributes of the IB learner profile, namely, knowledgeable, caring, inquirers, and open-minded. The IBLPQ was only developed based on four attributes of the IB learner profile that represent each construct in Bullock’s model [18], such as knowledgeable from the cognitive construct, caring from the affective construct, inquirers from the conative construct, and open-minded from the social construct. Besides, the selection of the four attributes was owing to the mission of IB to prioritize or focus on these attributes, as described in IB [31] (p. 1), as follows:

“*The International Baccalaureate aims to develop inquiring, knowledgeable, and caring young people who help to create a better and more peaceful world through intercultural understanding and respect.*”

The development of the IBLPQ involved five stages, namely, initial item construction, qualitative Delphi study, quantitative Delphi study, pilot study, and field study. In the first stage, a thorough and comprehensive library study was conducted to generate items that match the operational definitions for the attributes of knowledgeable, caring, inquirers, and communicators. The format and number of the initial items to be generated were also specified during this stage. As such, Walker et al. [12] had generated a total of 32 items (eight items for each attribute) during the first stage. In the second stage, two qualitative Delphi studies were conducted on 23 experts who consisted of administrators, teachers who teach IB, primary school teachers, high school teachers, and IBDP students in Asia-Pacific countries to identify the content validity of the IBLPQ items. Based on expert opinion and feedback, 32 items generated during the first stage should be improved and one item should be eliminated.

Subsequently, in the third stage, a quantitative Delphi study was conducted on 50 administrators and experienced teachers to identify the content validity of 31 items that were improved during the second stage of IBLPQ development. The administrators and teachers were required to assess the items based on a five-point Likert scale ranging from 1 = most inappropriate to 5 = most appropriate. However, only 32 administrators and teachers had assessed the items. Walker et al. [12] further analyzed the content validity of the 31 items using Lawshe’s content validity ratio, Aiken’s V coefficient, and confidence interval for the mean of a rating scale, and the findings showed that the overall 31 items obtained a content validity coefficient between 0.77 and 0.91, proving that the items have content validity.

Walker et al. [12] subsequently conducted a pilot study on 976 IBDP students in 18 Asia-Pacific schools, such as China, Hong Kong, Indonesia, Lao PDR, Singapore, the Philippines, and South Korea, to indicate the psychometric properties of the IBLPQ, particularly the validity and reliability of the IBLPQ constructs using confirmatory factor analysis. The findings showed that all items have factor loading values of more than 0.6, and the measurement model for the IBLPQ had achieved the specified fit index with CFI = 0.93, RMSEA = 0.07, SRMR = 0.04, and X^2^ = 1964.2, df = 344. Besides, Cronbach’s alpha values for all four attributes also supported the reliability of the IBLPQ constructs, namely, knowledgeable (0.92), caring (0.94), inquirers (0.91), and open-minded (0.92). In other words, the 31 IBLPQ items have good psychometric properties in terms of validity and reliability. The findings also showed that all items have a factor loading value above 0.7 as suggested by Tabachnick and Fidell [32].

Since the pilot study proved that the IBLPQ has good psychometric properties, Walker et al. [12] then conducted the fifth stage, which is a field study involving 758 IBDP students in Indonesia, Singapore, Thailand, and Vietnam. Two items from the caring construct and two items from the open-minded construct should be eliminated due to overlapping factor loadings. As such, only 27 items were finalized using confirmatory factor analysis, and the findings showed that the IBLPQ measurement model comprising all of these items achieved the specified fit index with CFI = 0.9, RMSEA = 0.07, SRMR = 0.04, and X^2^ = 1770.2, df = 344. Besides, the Cronbach’s alpha values for the overall four attributes further supported the reliability of the IBLPQ constructs, namely, knowledgeable (0.92), caring (0.94), inquirers (0.91), and open-minded (0.91).

Based on the pilot study and field study conducted by Walker et al. [12], the IBLPQ has shown good psychometric properties with high reliability and construct validity. In other words, all of the 27 items have met the fitness index, with the IBLPQ measurement model comprising the four constructs of knowledgeable, caring, inquirers, and open-minded.

## 3. Materials and Methods

### 3.1. Research Design

The study was based on a quantitative approach. The survey was selected as a research design, while online data collection using questionnaires was selected as the research instrument because it is easily administered and capable of collecting data in a detailed and organized manner [33,34].

### 3.2. Research Instrument

This study was conducted to develop the 10 IB learner profile instrument (10IBLP-I). In this study, the generation and development of items were based on Creswell [35], Fowler [36], Netemeyer et al. [37]. The first step after developing the conceptual framework of 10IBLP-I was the generation of items for measuring attributes as the IB learner profile constructs, which was acquired from the following sources: (1) a thorough and comprehensive literature review; (2) adaptation from the existing instruments comprising items from four attributes of IB learner profile developed by Walker et al. [12] and Bryant et al. [13]; and (3) interviews with field experts [37,38,39,40,41]. Each construct was also ensured to consist of at least three items to corroborate the construct as suggested by De Vellis [38]. The final step was the selection of a scale to measure the constructs of IB learner profile, particularly using a four-point Likert scale from 1 = “Strongly Disagree” to 4 = “Strongly Agree”. A middle point was not used in this instrument [42] because this may have allowed the respondents to answer without making a decision [43]. The scale is more appropriate compared to the conventional scoring method for the use of the Rasch model in this study. The item generation process produced a total of 114 items, as per Table 1, to be analyzed using the Rasch measurement model.

Before the questionnaires were distributed, approval to conduct research was applied by the Education Policy Planning and Research Division, MOE as well as involvement from the State Education Department. After that, a randomly designated list of students (based on the sampling framework) as well as the instrument built on the Google Drive platform was shared with the appointed teacher representatives to administer the study at their respective school levels. A period of one month from October to November 2020 was given to the teachers and students to complete the questionnaires.

The instrument had undergone the process of content validity through the content validity index (CVI) technique by 11 professional and field experts. Item review was conducted based on recommendations by the experts. Face validity for this instrument was reviewed by a lecturer, and an excellent Malay language teacher had also reviewed the aspects of vocabulary, sentence structure, and terminology used so that the instrument could be comprehended by the year four students. Subsequently, a total of 20 students were selected for face validation. They were assigned to identify and list out any incomprehensible vocabulary or terminology. Besides, they were also allowed to give an opinion to improve the quality of the questionnaires in terms of font size and design so that the questionnaires were more easily understood by the research sample.

### 3.3. Sampling

This study involves two phases that include a pilot study and a field study. A simple random sampling was used for the pilot study whilst disproportionate stratified random sampling was used for the field study. The target population of this study are 16 years old students and they are also year four students from the secondary school of IBWS MOE, with a total amount of 1422 students. The students were selected as a sample as they have gained four years of experience as IBMYP students and have developed a good understanding of IB learner profile. Moreover, the continuous application of standards and requirements among students that began since year one has enabled them to deeply appreciate the implementation of IBMYP and apply it in everyday life.

A pilot study was administered to 170 respondents of year four students of IBWS MOE schools; however, only 151 questionnaires (61 male students and 90 female students) could be used because 19 respondents did not answer the questionnaires. The number of respondents involved in the pilot study is sufficient based on the recommendation by Linacre [44], who specified a minimum requirement of 108 respondents for polytomous data based on a 99% confidence interval with a calibration value of ±0.5 logits to enable the analysis of Rasch measurement model to be implemented. Data obtained from this pilot study were analyzed using the Rasch measurement model to identify item fit, unidimensionality, item-individual map, reliability, and separation index for items and respondents.

Next, a field study was conducted to determine the construct validity based on the classical test theory (CTT) by using confirmatory factor analysis. A total of 460 respondents of year four students in 10 IBWS MOE schools were selected as the sample of this study as suggested by Hair et al. [45] for confirmatory factor analysis using a structural equation model. Disproportionate stratified random sampling was used to administer the field study in which 46 year four students from each IBWS MOE school were selected as the respondents of this study. The aim of using this technique is to provide equal opportunity for students as the number of schools for each category is different based on school type. There are three types of schools taken into account in this sampling, namely, daily secondary school, boarding school and religious high school. However, only 446 questionnaires (179 male students and 267 female students) could be analyzed to identify the construct validity of this instrument as 14 respondents did not answer the questionnaires. Overall, the response rate for the distributed questionnaires is 97%. This response rate is considered sufficient with more than 90 percent of the response rate and in line with past studies from Marret and Choo [46], and Alias and Nur Ain [47].

### 3.4. Statistical Analysis

Rasch measurement model was used to analyze the pilot study data. According to Linacre [48], this model can test and examine the psychometric properties of an instrument in validity and reliability aspects. WINSTEPS version 3.71.0 software (John M. Linacre Chicago, IL, USA) was used to analyze the following aspects of item functionality: (1) item fit based on the infit and outfit values in the range of 0.77 to 1.3 logits [49]; (2) item polarity based on the positive value of point measure correlation (PTMEA CORR); (3) local item dependency analysis; (4) unidimensionality based on principal component analysis of residual (PCA); (5) item-individual map that determines the positions of items and respondents on the same logits scale; and (6) reliability and separation index for items and individuals. The reliability value can be measured based on a good internal consistency value (Cronbach’s alpha), which is acceptable if it exceeds 0.7 [50]. Item separation index refers to a variety of item difficulty levels, while individual separation index refers to a variety of students’ ability levels in answering the questionnaires. An index value between 4.0 and 5.0 is categorized as very good [49].

The classical test theory using confirmatory factor analysis was also employed in this study to identify the construct validity of 10 IBLP-I. Confirmatory factor analysis is a statistical technique used to validate the factor structure for instrument measurement and is operated on using the measurement model using IBM SPSS analysis moment of structure (AMOS) 23 (IBM Corporation, Chicago, IL, USA). The appropriateness of a measurement model fitness is assessed based on the fit indices suggested by Hair et al. [45] and Holmes-Smith et al. [51]. The fit indices of a measurement model are assessed based on at least three categories of fit indices by entering at least one index from each category to achieve the fit model. The three categories include absolute fit, incremental fit, and parsimonious fit. In this study, the fit index used for the category of absolute fit was the root mean square of error approximation (RMSEA), while the category of incremental fit used comparative fit index (CFI) and Tucker–Lewis index (TLI), and finally, for the category of parsimonious fit, the fit index used was Chi-square/degrees of freedom ratio (Chisq/df). As stated by Hair et al. [45] and Holmes-Smith et al. [51], a model is deemed fit when TLI ≥ 0.90, CFI ≥ 0.90, RMSEA ≤ 0.08, and Chisq/df ≤ 5.0 as shown in Table 2.

## 4. Results

### 4.1. Psychometric Properties of 10IBLP-I Based on Rasch Measurement Model

A total of 114 items, based on 10 constructs of 10IBLP-I, were measured as per item fit, unidimensionality, reliability index, and separation index. In this stage, items that fit and contribute to good psychometric properties were retained, while misfit items were proposed to be reviewed or eliminated [54]. Besides, the Rasch measurement model can also be used to review the suitability of the Likert scale used in this study based on the six criteria specified by Linacre [55].

#### 4.1.1. Item Fit

Item fit refers to fit analysis for each item in the questionnaire with the Rasch measurement model [56]. In accordance with the Likert-scaled polytomous data in this study, the selected mean square MNSQ value was in the range of 0.77 to 1.3 [49]. Meanwhile, the productive Zstd value was in the range of −2.0 to +2.0 [57], and this value can be ignored if the value of MNSQ has been accepted [58]. The MNSQ value is measured based on both infit and outfit values, so that only the items that fit the model would be considered for the next analysis because the misfit items do not contribute to the measurement of constructs and are considered weak.

After observing the infit and outfit values of MNSQ, a total of 73 items were retained and the remaining were eliminated to increase the Rasch measurement model fit. The value was in the range of 0.78 to 1.31 logits. Observation on the standard error value for the data was in the range of 0.10 to 0.15, and this value refers to the element of accuracy in a calculation [58]. Fisher [49] considered the range of this error value sufficient. Besides, item fit can also be measured based on item polarity by measuring the PTMEA CORR value. This value refers to a group of items that measure the same construct, including the assumption that the items measure a single construct [57]. In this study, the PTMEA CORR value obtained was in the range of 0.35 to 0.66 and complied with the minimum value of 0.3 [59]. The positive PTMEA CORR value showed that the retained items could contribute to the psychometric properties of 10IBLP-I, and subsequently be able to discriminate the IBWS MOE students. The next item measurement aspect is local item dependency analysis.

The local item dependence is generally assessed based on the standardized residual correlation value between two items, with a maximum value of 0.3 [60]. If a pair of items has a correlation value of more than 0.3, only one item will be retained and another item will be eliminated from the model. The selection of the retained item will be based on the MNSQ value, which is approaching or equal to 1.0 [61], since this value is the expected value for the model fit [62]. This step is taken towards ensuring that the retained items do not overlap with other items [63]. Table 3 shows ten matching residual correlation values from 0.27 to 0.33. Items for the constructs of reflective and inquirers were retained to ensure that the number of items for each construct was sufficient to carry out confirmatory factor analysis at the field study level. This is because the relationship was still within the accepted range of 0.7 [61] and the pair of items were in the same construct.

#### 4.1.2. Eliminated Items

Items with MNSQ values exceeding 1.30 are known as under-fit items. These under-fit items were considered confusing, and examples of these items are as follows: E10, “*I can start a conversation with strangers*”; J11, “*I can control my feelings even under pressure*”; K11, “*I can express my feelings in a positive way such as reflection writing*”; and I10, “*I do not copy the work of others*”. These items might provide a different understanding to year four students. Item I10 might also be viewed as a negative item, while items J11 and K11 were more inclined towards the expression of feelings that might be somewhat odd and less practiced among students. Besides, items with MNSQ values of less than 0.77 are called over-fit items. These items are easier to predict and slightly disrupt the model fit. Among the items in the over-fit category are as the following: K02, “*I assess the suitability of the action taken*”; J03, “*I appreciate the importance of good mental health*”; C04, “*I strive to solve a problem even though it is difficult*”; and G01, “*I think of the best way to solve tough problems*”. Compared to the under-fit items, the overfit items are more easily expressed through actions as well as easily understood by students.

#### 4.1.3. Unidimensionality

Unidimensionality assumption compliance shows that a group of items in the developed instrument only measures a single construct [64]. Table 4 shows the value of principal component analysis of residual (PCA), which was recorded at 39.5% of variance explained by measures and found to be approaching model expectations of 39.6%, and was sufficient compared to the minimum value proposed by Reckase [65], which is 20%. Furthermore, unexplained variance in the first contrast was recorded at 3.7% and values below 5% are categorized as very good [49]. Additionally, the Eigenvalue for the reported variance was 3.3 and less than 5.0 [58], which shows no existence of a second dimension. Table 4 also shows that the unexplained variance in the first to the fifth contrast was between 3% to 5%, which is also categorized as very good [49]. Besides, the ratio rate between the variances explained by item size (17.9%) to the first component variance (3.7%) was 4.83, and exceeded the three-ratio minimum value [48].

#### 4.1.4. Reliability Index and Separation Index

The reliability index for 10IBLP-I is shown in Table 5 The Cronbach’s alpha value and item reliability are 0.96, and this value is categorized as excellent [49,50]. The reliability index for respondents or students in this study is 0.94, and this value is categorized as very good [49]. Moreover, according to Fisher [49], the separation index for items and respondents is also very good, which is between 4.0 and 5.0. This study did not consider the normality of the data prior to analysis, and the strata separation can be calculated based on the following formula:

(1) $$H=\frac{\left[\left(4\times separation\;index\right)+1\right]}{3}$$

Based on Table 5, the strata separation for respondents or students is 5.67, and this shows that the students’ capability levels can be categorized into five or six difficulty levels. Meanwhile, the strata separation for items is 6.6, and this shows that these items can be categorized into six or seven levels.

#### 4.1.5. Item-Individual Map

**Figure 1:**
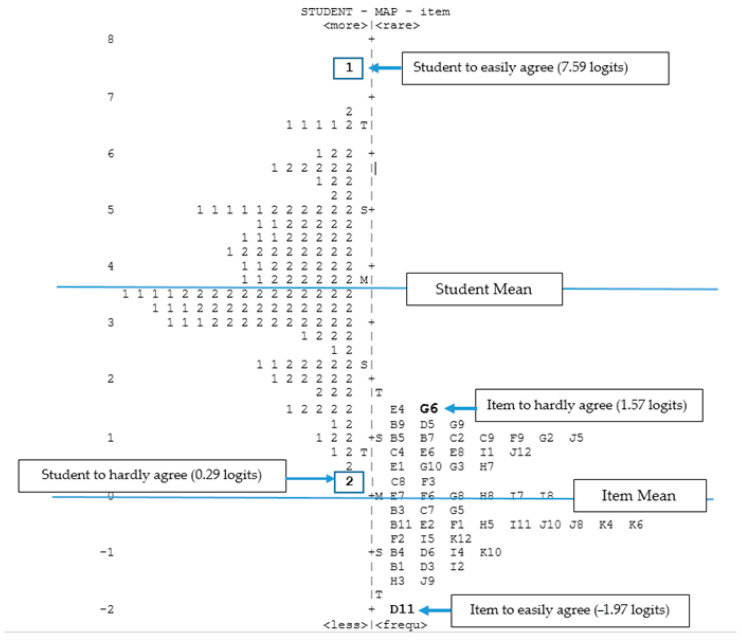
Item-individual map for 10IBLP−I.

Item-individual map is a graphical illustration that shows the distribution mapping of items and respondents involved in this study on a similar logits scale after undergoing the calibration process. In terms of instrument development or questionnaires, all of the items and respondents illustrated in Figure 1 below show various levels of difficulty agreement towards items and respondents’ agreement for each item [66]. This mapping is very useful because it can help researchers improve the psychometric properties of the developed instrument [66].

Based on Figure 1, the section on the left side of the logits scale represents the position of the respondents, namely, year four students, while the section on the right side depicts the arrangement of item difficulty levels. Overall, it was found that the mean of respondents (3.67 logits) has a higher position than the mean of items (0.00 logits). This shows that the items in 10IBLPQ-I are more easily agreed upon by the students.

The standard deviation values of items (−1.97 to +1.57) showed that the difficulty level measurement meets the range of +3.00 to −3.00, which is considered sufficient [67]. The highest student position arrangement was at +7.59 logits and the student is a male. The student was the easiest to agree with all items in the developed instrument. Meanwhile, the lowest position was presented by a female student with +0.29 logits, and this shows that the student was the hardest to agree with these items. The range of student agreement was 7.30 logits, whereas the range of item agreement was 3.54 logits. Furthermore, based on the item position hierarchy, item G06 (+1.57 logits) was the hardest to agree with by the students. Item G06 denotes “*I can answer questions critically*”, representing the construct of thinkers. The lowest item position was represented by item D11 (−1.97 logits) for the construct of open-minded. Hence, this item was the easiest to agree with by the students. Item D11 denotes “*I appreciate the favour of independence in Malaysia*”. This clearly shows that students can express their appreciation and respect towards their own culture and history.

#### 4.1.6. Scale Review

**Figure 2:**
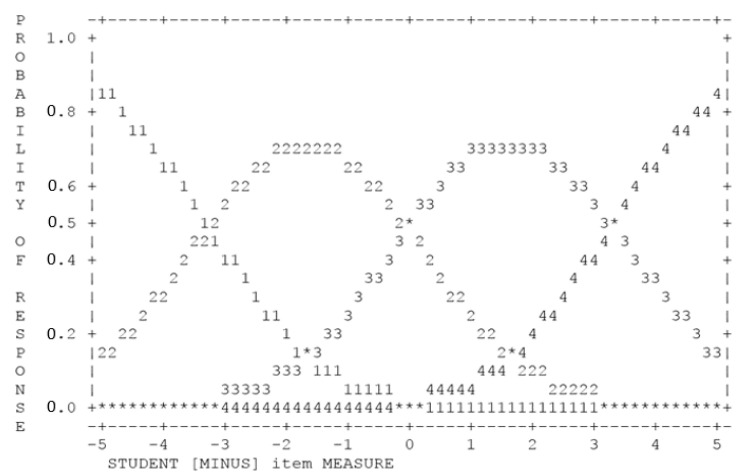
Probability curve for 10IBLP-I scale review.

The efficacy of the scale used in an instrument can be assessed using the Rasch measurement model, based on six specified criteria [55]. The first criterion is that each construct should have at least 10 observations and this criterion has been fulfilled in this study. As for the second criterion, each scale must show a probability curve peak as shown in Figure 2 below. The selection of a four-point Likert scale in this study has also fulfilled the third criterion, in which the average measure of each category increased in tandem with the level of scale with (1) 0.73 logits, (2) 1.48 logits, (3) 2.70 logits, and (4) 4.56 logits. This shows normal, uniform, and consistently increasing response patterns [63].

Next, all outfit MNSQ values were in the range of 0.94 to 1.22 and this fulfills the fourth criterion, in which the outfit MNSQ values should be less than 2.00 logits. As for the fifth criterion, the threshold values were arranged in an organized manner with −3.26, −0.02, and +3.28, and this shows no bias in the selection of any category of the scale used. The sixth criterion outlines that the restriction (difference value) for each scale should exceed the value of one, but less than five for a four-point Likert scale. A review was made for each restriction and it was found that the difference for each scale category exceeded the value of one, as follows:

S_1–2_ = 0.00 − (−3.26) = 3.26 (>1.0), S_2–3_ = −0.02 − (−3.26) = 3.24 (>1.0), S_1–2_ = 3.28 − (−0.02) = 4.00 (>1.0), (2)

### 4.2. Psychometric Properties of 10IBLP-I Based on Confirmatory Factor Analysis

**Figure 3:**
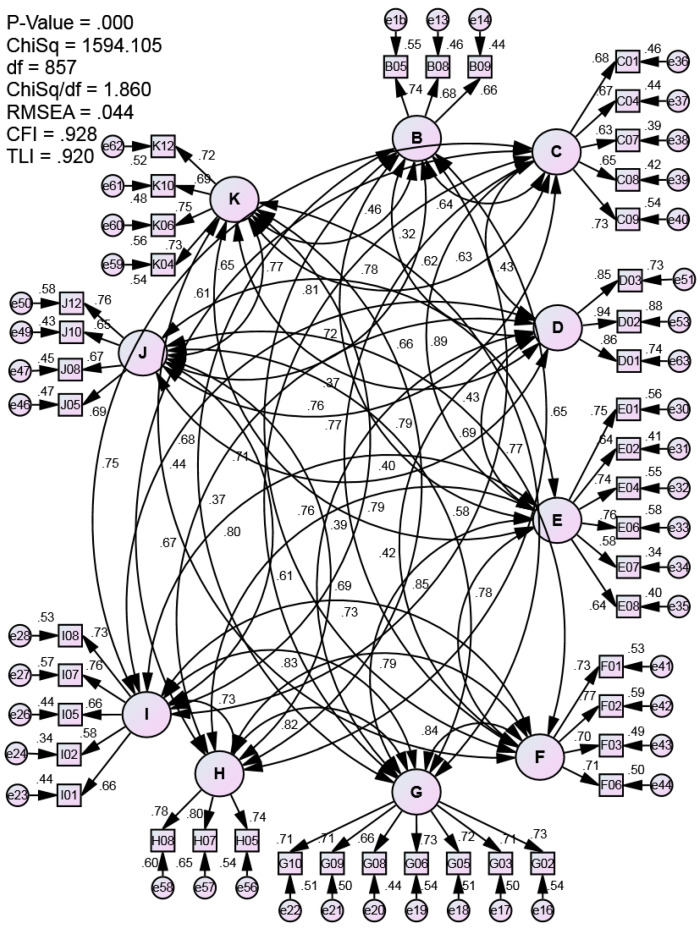
10IBLP-I measurement model. Note: B = caring, C = risk-takers, D = open-minded, E = communicators, F = knowledgeable, G = thinkers, H = inquirers, I = principled, J = balance, and K = reflective.

The 54 items analyzed using the Rasch measurement model were further analyzed using confirmatory factor analysis to determine the construct validity of 10IBLP-I. As a result, the fit indices values with CFI = 0.879 and TLI = 0.870 have not exceeded the specified fit indices threshold value of ≥ 0.9 [45,52]. In contrast to these, the RMSEA value of 0.049 was accepted because it did not exceed the specified fit index threshold value of ≥ 0.08 [45,53], including the value of χ^2^/df with 2.052, which was accepted because it did not exceed the specified threshold value of 5 [45]. Hence, 10 items have been eliminated one by one based on the factor loading below 0.5 [45] and modification indices more than 20 [68], which can improve the model fit for 10IBLP-I. It shows that only 19% of the total items have been eliminated and considered as minor modifications [45]. Figure 3 illustrates the measurement model, showing that the 44 items retained have factor loadings approaching or exceeding 0.6, as suggested by Hair et al. [45]. Although, the squared multiple correlation or R^2^ for items E07 and I02 is less than 0.4. However, according to Zainuddin [69], items that have an R^2^ less than 0.4 can be retained when the fit indices for the measurement model have met or achieved the fit indices.

Based on Figure 3 and Table 6, the fit indices values with CFI = 0.928 and TLI = 0.920 exceeded the specified fit index threshold value of ≥ 0.9 [45,52]. Similarly, the RMSEA value of 0.044 was also accepted because it did not exceed the specified fit index threshold value of ≥ 0.08 [45,53], including the value of χ^2^/df with 1.86, which was accepted because it did not exceed the specified threshold value of five Hair et al. [45].

Meanwhile, Figure 3 and Table 7 show the correlation values of each of the 10 attributes of 10IBLP-I. The correlations below 0.9 indicate adequate value to differentiate between each attribute.

Based on these findings, the 10IBLP-I measurement model has valid construct validity and item fit for the 44 items presented in Figure 3 and Table 8. The standardized factor loading for each item is in the range of 0.58 to 0.94. In addition, each attribute consists of at least three items and is considered adequate to measure the attributes or latent variables of 10IBLP-I [38,45].

## 5. Discussion

The findings discussed two main objectives, namely, (1) the development of the 10IBLP-I instrument to measure the level of IB learner profile among IBWS MOE students, and (2) to examine the 10IBLP-I psychometric properties based on the Rasch measurement model as well as confirmatory factor analysis. The combination of the CTT underlying the confirmatory factor analysis with the item response theory (IRT) for Rasch analysis is capable of producing robust and detailed validity and reliability compared to the study by Walker et al. [12], which tested the developed instrument based on the CTT only. Overall, the 10IBLP-I tested has good psychometric properties based on the 10 specified constructs; therefore, this study has filled the gap in the Walker et al. [12] study, which had only addressed four constructs. The final items in 10IBLP-I include a total of 44 items compared to 27 items in the IBLPQ.

Besides that, the development of this instrument has undergone a thorough process and taken into account the literature review and the views of IBWS MOE staff so that the constructed items meet the context of IBMYP practice in government schools in Malaysia. Therefore, this study also fulfills the gap related to the involvement of IBMYP students as the research sample, considering that most past studies have selected DP students as research samples, including Walker et al. [12]. Meanwhile, Bryant et al. [13] selected a sample of DP students with experience in IBMYP or PYP. However, this study did not use the Fuzzy Delphi method to generate items, as performed by Walker et al. [12]. Furthermore, the samples of the studies involved various countries in Southeast Asia, and comprised various races and religions compared to this study, which only used a homogenous research sample involving year four students from IBWS MOE who are mostly Malays. Besides, the sample size of this study was rather limited and this resulted in unsatisfactory construct validity analysis, with an average variance extracted (AVE) value approaching 0.50 [69]. In addition, both researches met all the requirements for model fit indices as an indicator for construct validity.

Other than construct validity, Walker et al. [12] also measured convergent validity and discriminant validity for the IBLPQ measurement model, which comprised all of the 31 items for the pilot study. The convergent validity of the items was measured using three methods, namely, factor loading value, average variance extracted (AVE), and composite reliability (CR). The findings showed that all items have a factor loading value above the specified threshold value of 0.7, as suggested by Tabachnick and Fidell [32]. Subsequently, the AVE value was measured using four constructs, namely, knowledgeable, caring, inquirers, and open-minded. The AVE value between 0.46 and 0.63 showed that the 31 items were in accordance with the constructs of knowledgeable, caring, inquirers, and open-minded. The composite reliability value also showed the same reliability value or above the specified threshold value between 0.7 and 0.91. Meanwhile, the discriminant validity for the IBLPQ measurement model was measured using four methods, namely, the square root of the AVE correlation value, Kenny’s mediation model with Chi-square statistics, Kenny’s mediation model with a model fit, and Anderson and Gerbing’s test. The findings of the discriminant validity analysis for all methods used showed that all four constructs have good discriminant validity.

Furthermore, the convergent validity of the IBLPQ measurement model for the 27 items was measured using three methods, namely, the factor loading value, AVE, and composite reliability. The findings showed that all of the items have a factor loading value above the specified threshold value of 0.7, as suggested by Tabachnick and Fidell [32]. Subsequently, the AVE value was measured using four constructs, namely, knowledgeable, caring, inquirers, and open-minded. The AVE value between 0.4 and 0.65 showed that all items were in accordance with the constructs of knowledgeable, caring, inquirers, and open-minded. The composite reliability value also showed the same reliability value or above the specified threshold value between 0.84 and 0.92. The findings of the discriminant validity analysis for the IBLPQ measurement model also showed that all four constructs have good discriminant validity.

Based on the comparison of AVE values between the constructs of caring (0.482), open-minded (0.782), knowledgeable (0.53), and inquirers (0.599), between 10IBLP-I and IBLPQ, the AVE value for this study is much better. Walker et al. [12] stated that the construct of inquirers obtained an AVE value of 0.40 compared to that in 10IBLP-I with a value of 0.599. This shows that 10IBLP-I has better psychometric properties in terms of convergent validity. However, similar to the findings by Walker et al. [12], the discriminant validity index in this study is poor because there was a correlation value between two constructs that exceeded the threshold value of 0.85 [70], namely, risk-takers and communicators. The correlational value between the two was 0.89 and this might be due to the confusion of students while answering the questions, considering that the operational definitions of the two constructs include the involvement of students in the collaborative network and teamwork spirit [6].

The reliability index analysis has been fulfilled at the levels of pilot and field studies based on (1) the item reliability index, respondent reliability index, and internal consistency index (Cronbach’s alpha) for Rasch analysis, and (2) composite reliability (CR) index and Cronbach’s alpha for the construct validity analysis. The findings for all reliability indices in this study were in line with the study by Walker et al. [12], which exceeded the threshold value of 0.70 [49,50]. Therefore, both 10IBLP-I and IBLPQ instruments have been proven to have a high level of consistency on most of the study samples.

Since 10IBLP-I was tested based on the Rasch measurement model, it is fair to address that this study has bridged the gap addressed in a past study, given that the IBLPQ was only developed based on the CTT Walker et al. [12]. The Rasch measurement model, founded by Georg Rasch in 1960, is a complete statistical method, and has the uniqueness of mathematical properties based on a parameter model that combines the difficulty level of items and the capabilities of the respondents, which further involves interactions between the two on a similar logit scale [62]. The selection of Rasch analysis at the pilot stage was due to its stability without being affected by the sample size [71]. Hence, the analysis results at the pilot level can help the researchers enhance the psychometric properties of the 10IBLP-I developed [48], before conducting a field study that involves a larger sample size. Besides, the Rasch measurement model can modify the four options on the Likert scale for each ordinal-scale data item to a scale in line with the size of the logits unit. In contrast to Walker et al. [12], who used a five-point Likert scale, the selection of the four-point Likert scale was more appropriate with this research sample and met all six criteria specified by Linacre [55]. As such, the students would not face any difficulty in making decisions that best represent them.

At the Rasch analytics stage, the calibration between students and their response to the items can determine the compatibility of each item constructed in the model, which subsequently prevents item repetition on the same measure [72]. Through precise testing, based on the MNSQ value in the range of 0.77 to 1.30 [49], the PTMEA CORR value, and local independence, only quality items are retained for the next testing. The local independence can also ensure that each constructed item only measures latent construct and do not overlap with each other [73]. This analysis also proves the unidimensionality of the developed instrument. Unidimensionality assumption compliance is a key aspect in the Rasch measurement model, assuming that the items in this instrument have a single capacity [42]. This is an early indication of good construct validity at the field study level.

### Research Limitations

There are several limitations to this study. The first limitation is that the 10IBLP-I was developed using the Malay language; hence, the instrument should be also translated to English so that it can be used in all IBWS around the world. The second limitation is related to the small sample size, as the target population of this study is year four students in IBWS MOE. The reason is due to the recognition of IBWS MOE, which in its fifth year and schools are still in the process of improving the IBMYP implementation based on IBO standards and requirements. The 10IBLP-I was also developed based on the standards and operational definitions specified by IBO, which explains why this instrument is more suitable to be used by the current IBWS. Hence, this is also a limitation for 10IBLP-I as it is applicable to IBWS only.

## 6. Conclusions

This article aims to identify whether the developed 10IBLP-I instrument has good psychometric properties based on the Rasch measurement model and confirmatory factor analysis. The study shows that 44 items meet the psychometric properties, with good validity and reliability. The findings also proved that a combination of the Rasch measurement model and confirmatory factor analysis can produce robust and consistent items with clear structures, and correspond to each construct. Overall, 10IBLP-I is an instrument that can be used to measure the level of IB learner profile among students who attend IBMYP either locally or overseas. In the research context, the development of 10IBLP-I by merging the analysis of the Rasch measurement model and confirmatory factor analysis has provided a theoretical implication in terms of empirical evidence related to the 10 attributes of the IB learner profile, namely, inquirers, knowledgeable, thinkers, communicators, principled, open-mindedness, caring, risk-takers, balanced, and reflective. Besides, the developed instrument to measure the level of IB learner profile among IBWS MOE students has empirical evidence of good psychometric properties, such as high validity and reliability. Accordingly, 10IBLP-I with good psychometric properties can be used by the Malaysia Ministry of Education to measure the level of IB learner profile among IBWS MOE students, to assess the effectiveness of IBMYP implementation of 10 IBWS MOE schools. Additionally, the use of 10IBLP-I in measuring the level of IB learner profile is not only limited to students of IBWS MOE, but also to students who attended IBMYP in private schools and MARA Junior Science College. In line with this recommendation, a further study can be carried out to identify the level of IB learner profile not only among the students of IBWS MOE, but also students who attend IBMYP in private schools and MARA Junior Science College, using 10IBLP-I and evaluation of the effectiveness of IBMYP implementation in the schools. Future researchers are also recommended to identify student profiles based on gender as well as types and school locations, by using 10IBLP-I. Moreover, 10IBLP-I can be adapted to the nine student profiles in the national curriculum so that it applies to other government secondary schools in Malaysia. This research also can be expanding with the measurement invariance analysis to further validate the instrument, such as differential item functioning (DIF), across demographic profile.

## Figures and Tables

**Table 1:** Number of items by construct.

Construct	No. of Items
Caring	11
Risk-Takers	11
Open-Minded	12
Communicators	12
Knowledgeable	11
Thinkers	11
Inquirers	11
Principles	11
Balanced	12
Reflective	12

**Table 2:** Fit Indices.

Fit Index	Name of Index	Fit Index Value	Source
Absolute fit	Chi-square/Degrees of freedom ratio (χ^2^/df)	χχ^2^/df ≤ 5	[45]
Incremental fit	Comparative Fit Index (CFI)	CFI ≥ 0.9	[45,52]
	Tucker–Lewis Index (TLI)	TLI ≥ 0.9	[45,52]
Parsimonious fit	Root Mean Square Error of Approximation (RMSEA)	RMSEA ≤ 0.08	[45,53]

**Table 3:** List of local items dependence.

Correlation	Item No.—Construct	Item No.—Construct
0.33	K04: Reflective	K06: Reflective
0.31	H05: Inquirers	H08: Inquirers
0.30	I02: Principled	I04: Principled
0.29	E01: Communicators	G06: Communicators
0.29	J09: Balanced	K06: Reflective
0.29	G09: Thinkers	G10: Thinkers
0.28	J05: Balanced	J12: Balanced
0.28	B05: Caring	B09: Caring
0.28	F01: Knowledgeable	F02: Knowledgeable
0.27	H03: Inquirers	H05: Inquirers

**Table 4:** Principal component analysis of residuals (in Eigenvalue unit).

	Empirical		Modeled	
Total raw variance in observations	89.2	100.0%		100.0%
Raw variance explained by measures	35.2	39.5%		39.6%
Raw variance explained by persons	19.3	21.6%		21.7%
Raw Variance explained by items	15.9	17.9%		17.9%
Raw unexplained variance (total)	54.0	60.5%	100.0%	60.4%
Unexplained variance in 1st contrast	3.3	3.7%	6.1%	
Unexplained variance in 2nd contrast	2.7	3.0%	5.0%	
Unexplained variance in 3rd contrast	2.6	2.9%	4.9%	
Unexplained variance in 4th contrast	2.5	2.9%	4.7%	
Unexplained variance in 5th contrast	2.4	2.7%	4.4%	

**Table 5:** Reliability index and separation index.

	Reliability Index	Separation Index	Strata Separation
Respondent	0.94	4.00	5.67
Item	0.96	4.70	6.60
Cronbach’s Alpha	0.96	-	-

**Table 6:** Model fit for 10IBLP-I.

Fit Indices	Fit Indices Threshold	Fit Indices Value for 10IBLP-I	Result
Absolute fit	χχ^2^/df ≤ 5	1.86 ≤ 5	Accepted
Incremental fit	CFI ≥ 0.9	0.928 ≥ 0.9	Accepted
	TLI ≥ 0.9	0.920 ≥ 0.9	Accepted
Parsimonious fit	RMSEA ≤ 0.08	0.044 ≤ 0.08	Accepted

**Table 7:** Correlations among 10 attributes of 10IBLP-I.

	B	C	D	E	F	G	H	I	J	K
**B**										
**C**	0.64									
**D**	0.32	0.43								
**E**	0.72	0.89	0.43							
**F**	0.61	0.77	0.42	0.78						
**G**	0.68	0.78	0.39	0.85	0.84					
**H**	0.65	0.65	0.37	0.76	0.80	0.82				
**I**	0.58	0.75	0.44	0.80	0.73	0.83	0.73			
**J**	0.62	0.76	0.40	0.81	0.69	0.79	0.69	0.71		
**K**	0.47	0.63	0.37	0.61	0.67	0.77	0.67	0.79	0.77	

**Table 8:** 10IBLP-I final items.

Constructs		Final Items	Factor Loading
B: Caring	B05	I care about the surrounding society.	0.74
	B08	I care about things going on overseas.	0.68
	B09	I take action to preserve the natural environment.	0.66
C: Risk-	C01	I am confident to try something new.	0.68
Takers	C04	I strive to solve a problem even though it is difficult.	0.67
	C07	I combine the idea of group members to obtain the best solution.	0.63
	C08	I persevered to complete the task even though it was difficult.	0.65
	C09	I have the courage to give a different idea from others.	0.73
D: Open-	D01	I respect the tradition of other races.	0.86
minded	D02	I respect the cultural value of other races.	0.94
	D03	I respect the cultural differences between races in my country.	0.85
E: Com-	E01	I can express an opinion on various things.	0.75
municators	E02	I listen to the opinions of others carefully.	0.64
	E04	I can interestingly convey my idea.	0.74
	E06	I always give my opinion when discussing in groups.	0.76
	E07	I can communicate politely.	0.58
	E08	I can adapt to all occasions or programs attended.	0.64
F: Know-	F01	I obtain information from a variety of sources.	0.73
ledgeable	F02	I obtain ideas based on various views.	0.77
	F03	I have different views on an issue after getting new evidence.	0.70
	F06	I apply the knowledge I have learned in my life.	0.71
G: Thinkers	G02	I solve a problem using a faster new method.	0.73
	G03	I analyse all information before making a decision.	0.71
	G05	I compare the options available before making a decision.	0.72
	G06	I can answer questions critically.	0.73
	G08	I always think first before giving an opinion.	0.66
	G09	I improve the existing material to a useful invention.	0.71
	G10	I have a solid reason for each decision that has been made.	0.71
H: Inquirers	H05	I use a variety of ways to obtain information about something.	0.74
	H07	I seek additional information after learning new things.	0.80
	H08	I explore new things in my learning.	0.78
I: Principled	I01	I am a person with integrity.	0.66
	I02	I think that integrity is an attribute that everyone should have.	0.58
	I05	I strive to be fair in my actions.	0.66
	I07	I can state the reasons for my principle.	0.76
	I08	I hold on strongly to my principle.	0.73
J: Balanced	J05	I can balance my learning time and leisure.	0.69
	J08	I maintain good relations with the community around me.	0.67
	J10	I spend time doing favourite activities with friends.	0.65
	J12	I can complete the task despite being active in the co-curriculum.	0.76
K: Reflective	K04	I consider the possible consequences for each decision that I take.	0.73
	K06	I always think about strategies to improve my weaknesses.	0.75
	K10	I re-think the things that I have said.	0.69
	K12	I evaluate every mistake that I have made.	0.72

## Data Availability

The data presented in this study are available on request from the corresponding author. The data are not publicly available due to the risk of identification of study participants.
